# Engineering Alginate-Based Dry Powder Microparticles to a Size Suitable for the Direct Pulmonary Delivery of Antibiotics

**DOI:** 10.3390/pharmaceutics14122763

**Published:** 2022-12-09

**Authors:** Beatriz Arauzo, Álvaro González-Garcinuño, Antonio Tabernero, Javier Calzada-Funes, María Pilar Lobera, Eva M. Martín del Valle, Jesus Santamaria

**Affiliations:** 1 Institute of Nanoscience and Materials of Aragon (INMA), CSIC—University of Zaragoza, 50009 Zaragoza, Spain; 2 Department of Chemical and Environmental Engineering, Campus Río Ebro-Edificio I + D, University of Zaragoza, 50018 Zaragoza, Spain; 3 Networking Research Center on Bioengineering, Biomaterials and Nanomedicine, CIBER-BBN, 28029 Madrid, Spain; 4 Department of Chemical Engineering, University of Salamanca, 37008 Salamanca, Spain

**Keywords:** alginate, microparticles, atomization, colistin sulfate, antimicrobial activity, lung, infection, *Pseudomonas aeruginosa*

## Abstract

The inhaled route is regarded as one of the most promising strategies as a treatment against pulmonary infections. However, the delivery of drugs in a dry powder form remains challenging. In this work, we have used alginate to form microparticles containing an antibiotic model (colistin sulfate). The alginate microparticles were generated by atomization technique, and they were characterized by antimicrobial in vitro studies against *Pseudomonas aeruginosa*. Optimization of different parameters allowed us to obtain microparticles as a dry powder with a mean size (Feret diameter) of 4.45 ± 1.40 µm and drug loading of 8.5 ± 1.50%. The process developed was able to concentrate most of the colistin deposits on the surface of the microparticles, which could be observed by SEM and a Dual-Beam microscope. This produces a fast in vitro release of the drug, with a 100% release achieved in 4 h. Physicochemical characterization using the FTIR, EDX and PXRD techniques revealed information about the change that occurs from the amorphous to a crystalline form of colistin. Finally, the cytotoxicity of microparticles was tested using lung cell lines (A549 and Calu-3). Results of the study showed that alginate microparticles were able to inhibit bacterial growth while displaying non-toxicity toward lung cells.

## 1. Introduction

Respiratory diseases caused by multidrug-resistant (MDR) Gram-negative bacteria (i.e., *Pseudomonas aeruginosa*) are very difficult to overcome, leading to high mortality and morbidity. This is becoming one of the main problems in global healthcare in the context of increasing resistance to antibiotics [1]. The classic treatment of this type of infection is carried out by systemic administration [2]. However, with the parenteral route, only a small fraction of the antibiotics reach the lungs, lowering their effectiveness and needing high doses [3]. As a consequence, the drug excess may be distributed to non-target organs, where it could cause adverse effects; among these, gastrointestinal symptoms or hearing impairment have been cited [4].

In contrast, the inhalation route presents significant advantages. In particular, inhaled antibiotic therapy can provide many benefits for the treatment of respiratory infections [5]. Therefore, the main advantage offered by this type of administration is that the drug directly reaches the lung (infection site) and, as a consequence, an optimal drug concentration and prolonged time action can be achieved using a lower dose, reducing the side effects and improving the quality of patient life comparing to other administration routes [6]. Colistin sulfate (CLS) is an effective antibiotic against Gram-negative strains. It belongs to the polymyxin group. It is a cationic lipopeptide antibiotic formed by two different parts, a cyclic decapeptide and a fatty acyl chain. This amphipathic structure facilitates its contact with the outer membrane of the bacteria [7]. It has a heavy binding affinity for lipopolysaccharide (LPS) and in the outer membrane, there are many targets. This interaction produces a displacing of Ca^2+^ and Mg^2+^ ions, the firmness of the outer membrane decreases and the permeability increases [8]. It promotes the disruption of the membrane and bacterial death because colistin sulfate molecules have a positive charge and lipopolysaccharides (LPS) are negatively charged [9]. The main drawback of its use is related to its nephrotoxicity when it is administrated by intravenous route. These side effects can occur in up to 60% of patients, which limits the dose of this antibiotic used [10]. Preliminary studies have shown that inhaled colistin sulfate increased lung function and reduces inflammatory parameters [11]. Furthermore, previous studies have described that the amorphous powder form of this drug is able to absorb a high quantity of water when it has been exposed to a high relative humidity [12,13]. Therefore, the administration of crystals is desired in order to reduce the mentioned hydration processes and the subsequent aggregation of the deliquescent particles.

It seems clear that it is needed to develop new formulations in order to achieve a direct pulmonary delivery. Wet nebulization is the most common way to administrate antibiotics by pulmonary route; although, it presents many disadvantages including complex administration procedures, more susceptibility to reinfection, drug instability and less acceptance by patients compared to the Dry Powder Inhalers (DPI) [14]. Because of this, biodegradable polymeric microparticles loaded with antibiotics are being investigated as a dry powder to be administrated with DPI [13,14].

Among the natural polymers, alginate has been used for decades in the pharmaceutical and biomedical industry as a carrier of drugs to different parts of the body. This polymer has a natural origin and shows no toxicity, biodegradability or high biocompatibility [15]. One of its most interesting properties is the capacity to form gels when crosslinked with divalent cations (e.g., Ca^2+^, Ba^2+^, Zn^2+^) [16,17]. These gels are formed due to an ionic interaction process, where a high biocompatible matrix is formed that allows the retention of many different types of drugs [18], proteins [19] and even, living cells [20]. Among the most used techniques to generate alginate microparticles, emulsion and spray drying can be highlighted [15,16]. They both allow the generation of particles of adequate size; although, it is important to note that emulsion-based processes often involve many steps and the use of organic solvents, which could affect the biocompatibility of the microparticles after their administration [21]. On the other hand, spray-drying is cost-effective and amenable to continuous production. Previous articles [22] have prepared alginate microparticles in a dry powder form using spray drying, but the resulting material had a very broad size distribution. In addition, it is important to take into account that this technique requires drying at high temperatures, which may compromise the functionality of the drug or of the polymer used. Finally, atomization in droplets over a cationic solution produces highly monodisperse microparticles with a high yield. However typical sizes are even larger, often in the size range 40–60 µm in water. The particles obtained with this process are required to be dried [23].

In summary, the production of drug-loaded cross-linked alginate microparticles with adequate size for pulmonary delivery (0.5–5 µm) is still a challenge. Several inhaled formulations have been designed to deliver colistin sulfate onto the lung epithelium, such as liposomes [7] and microparticles prepared by spray drying [24] alone or in combination with other drugs [10]. Therefore, the chief objective of this work is to develop a new formulation of colistin sulfate dry powder for inhalation based on alginate microparticles with a size < 10 µm. These particles were obtained after loading colistin sulfate on alginate beads that have been produced by an optimized atomization procedure using barium chloride as a crosslinker in the receiving solution. This is remarkable considering the large sizes that usually result from using this procedure. Colistin sulfate particles were mainly deposited on the outside of the alginate microparticles, a feature that would allow the fast distribution of the drug once the site of action has been reached. Cytotoxicity experiments were developed in two lung epithelial cell lines, A549 (alveolar) and Calu-3 (bronchial), that confirmed the biocompatibility of microparticles with cells of the respiratory tract. Finally, antimicrobial activity was evaluated against one of the most common bacteria responsible for pulmonary infections, *Pseudomonas aeruginosa*.

In summary, this work presents a different production technique (atomization rather than spray drying) that allows good control of the particle characteristics and of the distribution of the active component. The resulting particles constitute a novel drug delivery system in dry powder form in an alginate matrix. In addition, the particles produced with the CLS antibiotic do not have excipients in the formulation and present a size suitable for inhaled administration.

## 2. Materials and Methods

### 2.1. Materials

Alginic acid sodium salt from brown algae (medium viscosity), calcium chloride (<99%) and phosphate-buffered saline (PBS) tablets (pH 7.4) were acquired from Sigma-Aldrich (Burlington, VT, USA). Dehydrated barium chloride (>99%) was supplied by Scharlau (Barcelona, Spain). Absolute ethanol was bought in PanReac (AppliChem, Darmstadt, Germany). Colistin sulfate salt (CLS) used was from MP Bio-medicals Laboratories (San Diego, CA, USA). Air Liquid (99.99%) (Paris, France) provided the air used in the atomization system.

Tryptone soy broth (TSB) and tryptone soy agar (TSA) were obtained from Conda-Pronadisa (Madrid, Spain). Eagle’s Minimum Essential Medium (EMEM) was purchased from ATCC (Alicante, Spain). Dulbecco’s modified Eagle’s medium (DMEM with stable Glutamine), Dulbecco’s Phosphate Buffered Saline (DPBS), penicillin, streptomycin and amphotericin B were obtained in Biowest (Nuaillé, France). Fetal bovine serum (FBS) was supplied by Thermo Fisher Scientific (Waltham, MA, USA).

The bacteria strain, *Pseudomonas aeruginosa* was bought in ATCC (ATCC 10145; Ielab, Alicante, Spain). Regarding cell lines, A549 (ATCC–CCL–185) (passages 35–40) was kindly gifted by Dr. P. Martin-Duque and used between and Calu-3 (ATCC-HTB-55) (passages 24–30) was acquired in ATCC.

### 2.2. Atomization Device and Production of Inhalable Powder Formulation

**Scheme 1:**
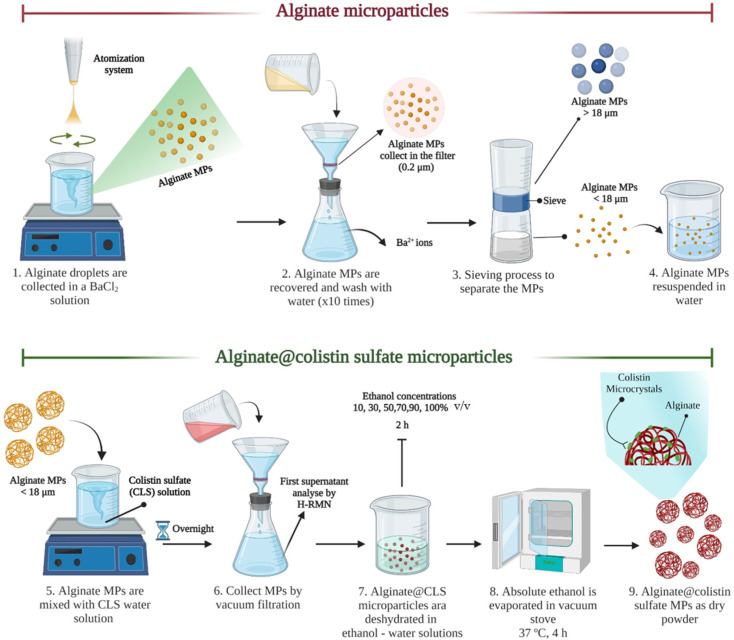
Protocol to obtain alginate microparticles (ALG MPs) loaded with colistin sulfate as dry powder.

An overview of the procedure is provided in Scheme 1. Alginate solutions (2% *w*/*v* and 2.5% *w*/*v*) were obtained from sodium alginate salt in distilled water. The polymer solution was kept under stirring (500 rpm), for 12 h at room temperature (RT). At the same time, barium chloride (Ba_2_Cl_2_) solution (2% *w*/*v*) was prepared in distilled water.

Microparticles were generated using atomization equipment, which was previously described by Herrero et al. [25]. The optimization process to obtain microparticles was adapted from that described in Arauzo et al. [26]. Briefly, the atomization system consists of a pressurized tank where alginate solutions are introduced. Air and liquid pressure values could be regulated, as it is important to note that the liquid pressure must be greater or equal to the air pressure. The optimal pressure values used for air and liquid were 0.9 bar and 1.0 bar, respectively. The flow-focusing nozzle has two differentiated parts, which are described previously by Cervero et al. [23]. One of them allows the entry of liquid polymer solutions (diameter of 300 µm) and the other (diameter of 350 µm) facilitates the entry of air (according to the established pressures values). Air provides enough kinetic energy to break the liquid jet, generating alginate droplets that are collected on a solution of BaCl_2_ under soft stirring (150 rpm). When the atomization process is completed, alginate microparticles were collected by vacuum filtration system (cutoff: 0.2 µm). Finally, with the objective to eliminate the residual Ba^2+^ ions, the microparticles were washed with water, this process was repeated 10 times. Finally, microparticles were subjected to a sieving process (sieve size < 18 µm, (C.I.S.A., Barcelona, Spain)). The microparticles that passed through the sieve were suspended in distilled water.

For drying, microparticles were filtered in a vacuum through a nylon membrane (0.2 µm) and successively dehydrated by washing with ethanol–water solutions (ethanol concentrations increased from 10 to 100% *v*/*v*). Microparticles were kept at each concentration for a minimum time of 2 h, until finally being suspended in absolute ethanol. At the end, absolute ethanol was evaporated in a vacuum stove (Memmert, Schwabach, Germany), 37 °C, 4 h. Dry alginate microparticles were stored at 4 °C.

The aqueous solution of alginate microparticles was mixed with a CLS water solution. CLS was dissolved in water in a proportion drug mass/polymer mass ratio of 35% *w*/*w*. The drug was in excess to promote its incorporation into the microparticles. The CLS was in contact with microparticles overnight, and after that, microparticles were recovered by vacuum filtration. Alg@CLS MPs were dehydrated and the first supernatant was collected to be analysed by H-RMN (more details in Section 2.7) in order to quantify the amount of CLS not anchored to microparticles.

### 2.3. Rheological Experiments

An oscillatory analysis was performed on the alginate solutions and the alginate-barium chloride systems to follow the crosslinking process by studying the variation in the storage modulus. A rheometer AR 1500 ex (TA Instruments, New Castle, DE, USA) was used to carry out the experiments. All the tests were performed with the same geometry (an aluminum plate of 4 cm in diameter with a 1 mm gap).

The rheological behavior of alginate solutions and alginate-barium chloride hydrogel were studied always at 25 °C, with a frequency sweep from 0.5 to 500 rad·s^−1^ at a fixed strain, that lies in the linear viscoelastic region. This strain was designated with a strain sweep analysis (from 0.5% to 300% strain) at an angular frequency of 1.0 rad·s^−1^, again at 25 °C. Based on the strain sweep results, the 1% strain was selected for the oscillatory analysis.

### 2.4. Size and Zeta Potential Measurement

Microparticles were characterized to obtain the particle size distribution (Scanning Microscope Electron (SEM), Cryogenic Dual Beam-Nova model 200 (Dual-Beam) and optical microscope), elemental analysis (Energy-dispersive X-ray spectroscopy (EDX)) and the surface charge distribution (Zeta potential).

Image description of the microparticles was carried out with a Leica DM1000 optical microscope (Leica Microsystems, Wetzlar, Germany), which is attached to a Leica DFC280 camera. In addition, the Measure IC software was used to estimate the size of the particles from the images obtained from this camera.

On the other hand, the size distribution of atomization microparticles after the drying process was assessed by SEM (voltages between 5–10 kV) and Dual-Beam with EDX. The last one was used at a voltage of 30 kV and currents of 0.1 nA and 50 pA. Each sample was covered with Palladium (Pd) (Leica EM ACE200 coater) for SEM or with Carbon (C) when they were analyzed with Dual-Beam and EDX. The Dual-Beam instrument was employed to cut the microparticles across an equatorial plane. Then EDX analysis was employed to obtain the distribution of CLS.

The SEM images were used to obtain the primary size distribution of the particles. To this end, the ImageJ software was employed, allowing the average size of the particles to be obtained. To ensure adequate statistical sampling, at least 4 different SEM images were used and three different regions of each sample were observed.

With the measurements of size, the main value obtained was the Span. Span gives an indication of how far the 10% (Dv10) and 90% (Dv90) points are apart from the midpoint (50%; (Dv50)) and it is defined in Equation (1):

(1) $$\mathrm{Span}\;=\frac{\mathrm{Dv}90-\mathrm{Dv}10}{\mathrm{Dv}50}$$

Zeta potential (laser Doppler anemometry) values of raw CLS and alginate microparticles (ALG MPs) were obtained using a Zetasizer Nano ZS^®^ (Malvern Instruments, Malvern, UK). Samples were suspended in a mixture of water and 1 mM KCl, sonicated and finally measured in the electrophoretic cell.

### 2.5. Powder X-ray Diffraction (XRD)

The crystallinity of the raw material and microparticles were determined by Powder X-ray diffraction (Empyrean, Malvern Panalytical, Malvern, UK). D/tex ultra-detector and Cu-Kα radiation sources were used. The samples were spread on a glass slide and located in the evaluating chamber. XRD scanning was set as 5 to 40° 2Ɵ at 0.013/200 s with a voltage of 45 kV and a current of 40 mA.

### 2.6. Solid State Fourier Transform Infrared Spectroscopy (FTIR)

Vertex-70 FTIR spectrophotometer (Bruker, Billerica, MA, USA) was used to analyze the raw material (colistin sulfate and alginate), the microparticles obtained after atomization (with and without antibiotic load) and confirm the possible interactions created between the antibiotic and the polymer in the final particles. It was completed with an attenuated total reflectance (ATR) sample stage. About 2.5 mg of each sample was deposited in the stage and each spectrum was obtained with a resolution 4 cm^−1^ in the range of 600–4000 cm^−1^ and 40-scan.

### 2.7. Drug Quantification

#### 2.7.1. Encapsulation Efficiency of Microparticles (% EE) and Drug Loading (% DL)

The drug content in microparticles was analyzed by H–NMR. Once the encapsulation process had finished, particles were collected by filtration as described in Section 2.3, the supernatant liquid was collected and the amount of CLS was measured by NMR using the spectrometer Varian 400 (Bruker, Billerica, MA, USA). 1H NMR was recorded at room temperature using Avance NEO 400 MHz (Bruker, Billerica, MA, USA) with a Prodigy CPPBBO BB-H&F z-gradient cryo-probe spectrometers (NMR Service, NUCLEUS, University of Salamanca, Salamanca, Spain). Chemical shifts (δ) are given in ppm with the solvent indicator as internal standard unless otherwise stated (CHCl3 7.26 ppm for 1H NMR; CH3OD 3.31 ppm for 1H NMR; DMSO 2.50 for 1H NMR). From the residual amount in the liquid, it is possible to calculate the amount of CLS in the particles. From these data, encapsulation efficiency (% EE) and drug loading (% DL) can be calculated by using Equations (2) and (3):

(2) $$\%\;\mathrm{EE}\;=\frac{\mathrm{CLS}\;\mathrm{mass}\;\mathrm{in}\;\mathrm{particles}}{\mathrm{CLS}\;\mathrm{mass}\;\mathrm{available}\;\mathrm{in}\;\mathrm{solution}}$$

(3) $$\%\;\mathrm{DL}\;=\frac{\mathrm{CLS}\;\mathrm{mass}\;\mathrm{in}\;\mathrm{particles}}{\mathrm{Particles}\;\mathrm{mass}}$$

#### 2.7.2. In Vitro Drug Release

The in vitro release studies of ALG@CLS MPs as a dry powder was carried out in PBS. The microparticles were incubated in 1 mL of solvent at 37 ± 1 °C (Thermo-Shaker, Biosan, Riga, Latvia) under shaking at 250 rpm. Samples were taken at fixed times (5, 15, 30, 45, 60, 120, 180, 240 min) and centrifuged (12,500 rpm, 5 min, RT). The pellet obtained from centrifugation was dispersed in 1 mL of fresh PBS. The supernatant was collected and analyzed by UPLC.

#### 2.7.3. Ultra-Performance Liquid Chromatography (UPLC)

A Water ACQUITY system H-Class was used to determine the concentration of CLS. This equipment consists of a thermostatic column with an autosampler, a binary pump and a photodiode array detector. A mass spectrometer (single quadrupole) with ionization by electrospray (ACQUITY QDa) is attached to the previous system. MASSLYNX software 4.1. (Water Corporation, Milford, CT, USA) was used for processing data (date: 10 November 2021). The required chromatographic separation (PREMIER HSS T3 UPLC column (1.8 µm, 2.1 × 100 mm, ACQUITY)) was performed at 80 °C with a mobile phase (70% acetonitrile and 30% Milli-Q water) that was modified with solution (0.1%) of formic acid, working at isocratic flow (0.5 mL/min).

CLS absorbance was monitored with a photodiode array detector (210 nm) and CLS was also quantified by mass spectroscopy using the most common ion (with an *m*/*z* ratio of 578.6). The retention time was 0.45 min for CLS.

A calibration curve of this drug was acquired using commercial standards of CLS (r^2^ > 0.990) within the necessary concentration range (0.25–20 µg/mL) in Milli-Q water. In the previous sample injection in the UPLC, the standards and samples were processed with a Nylon filter of 0.22 µm. Quantitative analysis was carried out in triplicate. The limit of detection (LOD) and the limit of quantification (LOQ) [27,28] were, respectively, 0.373 and 1.244 ppm for the conditions and analytical method employed.

### 2.8. Drug Release Modelling

Once release studies have been performed, their results were used for parameter estimation in order to model the CLS release behavior of these microparticles. The presence of CLS only on the surface (see Section 3.2) suggested not to use the traditional models that includes the diffusion inside the particle and/or an erosion process on the surface, as Pepas–Salin [29] or Higuchi [30] models. This particular distribution of CLS achieved in this work suggested that dissolution models could be more suitable to describe drug release. Particularly, the Noyes–Whitney model was performed for modelling experimental data, as a reference model in pharmacokinetics, summarized in Equation (4), representing the evolution of the concentration of the drug in bulk depending on time:

(4) $$\frac{dC}{dt}=k\cdot S_{w}\cdot \left(C_{\infty }-C\right)$$

where *C* represents the concentration of the drug, *C*_∞_ is the maximum concentration released, and *k* and *S_w_* are the intrinsic dissolution rate and the area of the cross-section of the colistin crystal (see Section 3.2), respectively. Therefore, *S_w_* is a function of time because the radius of the crystal is reduced as dissolution progresses. To estimate the area of the crystal (*S_w_*) the cross-sectional area of a sphere is used as an approximation, Equation (5):

(5) $$S_{w}=4\pi \cdot r^{2}$$

The variation of radius (*r*) along time is explained by the Aaron’s equation for spherical precipitate dissolution [31], shown in Equation (6):

(6) $$\frac{dr}{dt}=-m\cdot \frac{D}{r}-m\sqrt{\frac{D}{\pi \cdot t}}$$

where *D* is the colistin diffusion coefficient in the medium and *m* is an experimental constant.

These equations were implemented in gPROMS Model Builder 7.1. (PSE) as well as the experimental data obtained. Three parameters (*k*, *D* and *m*) were determined using hessian estimation with the heteroscedastic variance model (variance with a maximum 15% of the reference value). The accuracy of the model proposed was evaluated by two tests (goodness of fit and lack of fit test). The model was executed in a personal computer Intel Core i3 3.70 GHz and 4 GB RAM memory.

### 2.9. Antimicrobial Activity

Antimicrobial activity was observed in the Gram (−) bacteria model, *P*. *aeruginosa*. Raw colistin sulfate was used to establish the minimum inhibitory concentration (MIC) and minimum bactericidal concentration (MBC) values. Therefore, ALG@CLS MPs were tested at the same colistin sulfate concentrations.

*P*. *aeruginosa* was grown for 16 h in TSB in a shaker Innova^®^ 40 (New Brunswick Scientific, Enfield, CT, USA) 150 rpm and 37 °C. Lastly, 10^8^–10^9^ colony-forming units/mL (CFU/mL) were obtained. Experiments were made with a final concentration of ~10^5^ CFU/mL. Different concentrations of raw CLS and ALG@CLS MPs (from 0.125 to 8.00 µg/mL) were inoculated with *P*. *aeruginosa* in order to reach the MIC and MBC values.

#### 2.9.1. Optical Density (OD600) Measurements

Optical densities of bacteria were analyzed at 600 nm (Implen^TM^ OD600 DiluPhotometer^TM^; ThermoFisher Scientific, Waltham, MA, USA) in order to monitor its growth. *P*. *aeruginosa* with an initial concentration of ~10^5^ CFU/mL was added to the samples. These mixed suspensions were maintained in a shaker at 150 rpm and 37 °C. The optical density was measured during 24 h at different times.

#### 2.9.2. Agar Dilution Method

Bacterial cultures were diluted up to ~10^5^ CFU/mL. Different concentrations of microparticles were used to treat the bacterial cultures. After that, the bacteria were kept in a shaker for 24 h (37 °C, 150 rpm). Subsequently, bacteria suspensions were diluted in PBS and Petri plates with TSA were used to seed the different bacterial solutions. Petri plates were kept at 37 °C for 24 h before measuring the number of CFU/mL values. The MBC was established as the concentration that exhibited no visible bacteria growth, i.e., the lower concentration employed that is capable of killing > 99.95% of the bacteria).

Bacterial cultures were diluted up to ~10^5^ CFU/mL and then inoculated with different concentrations of microparticles. Bacteria were kept in a shaker at 37 °C at 150 rpm for 24 h. Then, bacteria suspensions were diluted in PBS and seeded in Petri plates with TSA at 37 °C for 24 h before measuring the number of CFU/mL values. The MBC was determined by testing the concentration that exhibited no visible bacteria growth (the lowest concentration that kills > 99.95% of the bacteria).

#### 2.9.3. SEM Analysis

Changes in bacterial surface morphology were directly observed by SEM to confirm the efficacy and antimicrobial activity of CLS in this bacterial model. This analysis required a previous fixation process in order to maintain bacteria properties unaltered after the incubation experiments. After 24 h of contact of bacteria cultures with raw colistin sulfate and ALG@CLS MPs cultures to be observed by SEM. Briefly were centrifuged at 3000 rpm, 5 min at RT (Micro Star 17R, VWR, Radnor, PA, USA). The pellet was collected, washed twice with 0.1 M PBS and fixed in 2.5% (*v*/*v*) glutaraldehyde overnight. Then, the sample was washed twice with distilled water, to eliminate the glutaraldehyde. The pellet was suspended in water, the liquid was filtered through a nylon membrane 0.2 µm (Millipore, Dublin, Ireland) and, subsequently, it was dehydrated in a series of absolute ethanol washes (10, 30, 50, 70, 90 and 100% ethanol *v*/*v*) for 15 min at each concentration. Finally, absolute ethanol was allowed to evaporate and the samples were sputtered with Pd prior to SEM analysis.

### 2.10. Cytotoxicity Studies

#### 2.10.1. Cell Culture Conditions

Cell line A549 (alveolar epithelium cells) were cultured to confluence in 100 mm Petri Dishes at 5% CO2, 37 °C in Dulbecco’s Modified Eagle´s Medium (DMEM; Gibco, Invitrogen, Waltham, MA, USA). Calu-3 cells, human bronchial epithelium, were cultured to confluence in 25 cm^3^ flasks at the same conditions as A549 in Eagle´s Minimum Essential Medium (EMEM; ATCC, Barcelona, Spain). Both culture mediums were supplemented with 10% *v*/*v* fetal bovine serum (FBS). A mixture of antimitotic–antibiotic was added to the medium, penicillin (60 µg/mL), streptomycin (100 µg/mL) and amphotericin B (0.25 µg/mL).

#### 2.10.2. Alamar Blue (AB) Assay

The AB assay was performed to estimate the effect of alginate microparticles with and without CLS on cell viability in comparison with free colistin sulfate. This test allowed us to identify the highest concentration of microparticles (and antibiotic) that exhibited toxicity towards A549 and Calu-3 cells. 1 × 10^4^ cells/well in 100 µL were seeded onto 96 well plates and allowed to attach for 24 h in a CO_2_ incubator. Raw colistin sulfate and ALG@CLS MPs were put in contact with a culture medium at concentrations of 0.1 μg/mL to 100 μg/mL. Cells incubated in a culture medium without particles were set as a positive control. Control and sample cells were incubated (at 37 °C, 5% CO_2_) for 24 h and 48 h. The medium (with and without samples) was removed and DPBS was used to wash cells (this process was repeated twice). Subsequently, the cells were incubated with a fresh medium mixed with the AB reagent (10% *v*/*v*). The conditions of the experiments were determined following the manufacturer’s instructions (incubation time > 1 h at 37 °C and 5% CO_2_). The fluorescence displayed was measured at ʎ530 nm excitation and ʎ590 nm emission using a microplate reader Multimode Synergy HT (Microplate Reader; Biotek, Winooski, VT, USA). Equation (7) was used to calculate the cell viability, where MFV is the mean fluorescence value:

(7) $$\%\;\mathrm{Cell}\;\mathrm{viability}\;=\frac{\mathrm{MFV}\;\mathrm{of}\;\mathrm{treated}\;\mathrm{cells}}{\mathrm{MFV}\;\mathrm{of}\;\mathrm{control}\;\mathrm{cells}}\times 100$$

The ISO 10993-5 standard was established to define the toxicity limit (Biological evaluation of medical devices—Part 5: Tests for in vitro cytotoxicity), which admits a material as non-cytotoxic if the cell viability value obtained is >70%.

## 3. Results and Discussion

### 3.1. Rheological Analysis, Atomization and Concentration Effect in Microparticles Generation

**Figure 1:**
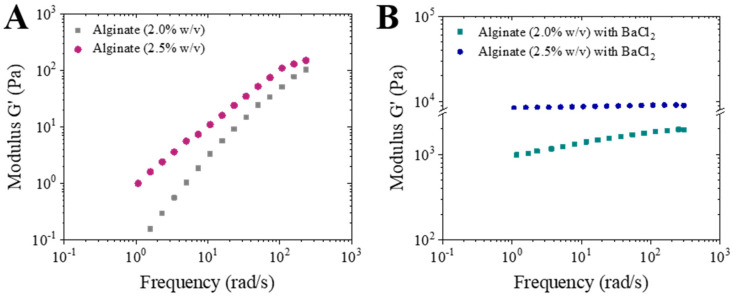
Rheological analysis of: (**A**) Alginate solutions (2% and 2.5% *w*/*v*); (**B**) Alginate gels (2% and 2.5% *w*/*v*) with BaCl2 (2% *w*/*v*) as crosslinker.

The rheological analysis of alginate solutions and alginate gels is shown in Figure 1. It shows the results, in terms of the storage modulus (G’), as a function of the applied frequency for the different alginate solutions and the respective hydrogels formed after crosslinking. As can be observed, there is a great difference between the rheological behavior of the alginate solutions and the alginate gels, and a jump of several orders of magnitude in the value of G’ can be observed, indicating that crosslinking was successfully performed. The mechanical spectra of the alginate solutions are typical for polymeric solutions. However, the G’ of the hydrogels, after solutions crosslinking, is independent of the frequency. This rheological behavior is expected for hydrogels. Moreover, the storage modulus of the gels that were obtained from a more concentrated alginate solution is higher because there are more groups that can be crosslinked with the barium chloride. This phenomenon has been previously reported by Arauzo et al. [26]. Atomization was the technique selected and BaCl_2_ has been used as cross-linker due to the ability to form more resistant spheres in contrast with calcium chloride [32].

Taking into account the above results, conditions for atomization were fixed as 1.0 and 0.9 liquid and air pressure, respectively [26]. Alginate at 2.5% *w*/*v* concentration was atomized with these pressures in order to reduce the particle size, since the results obtained showed that the highest alginate concentration allowed it to generate smaller particles (Table S1 in Supplementary Materials). The spherical morphology of ALG MPs could be observed by optical and electron microscopes (Figure S1).

### 3.2. Characterization of Microparticles

**Figure 2:**
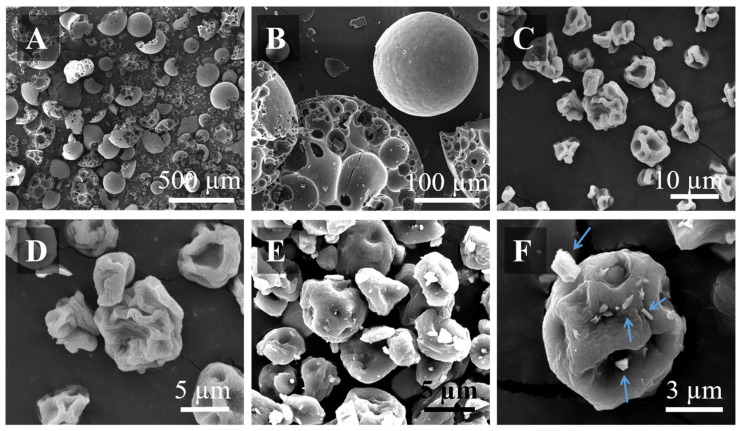
Images obtained by Scanning Electron Microscopy (SEM) of: (**A**,**B**) Raw Colistin Sulfate; (**C**,**D**) Alginate microparticles; (**E**,**F**) ALG@CLS MPs. CLS crystals are visible on the surface of the alginate microparticles (some are marked by blue arrows in Figure 2F).

The generated alginate-based microparticles were dried (as defined in Materials and Methods, Section 2.2) the SEM analysis confirmed the presence of microparticles after the drying process. The particles had a pseudo-spherical morphology and rough surface due to the process of dehydration using increasing ethanol concentrations and drying in the vacuum stove (Figure 2C–F). The particle size distribution was very similar for alginate-based microparticles, without and with CLS loaded on them, 4.80 ± 1.60 µm and 4.45 ± 1.40 µm, respectively. This size would be suitable for pulmonary drug delivery if the particles can be aerosolized in a disaggregated form. At the same time, it is worth emphasizing that the drying process was able to reduce particle size four times in comparison with initial particles in water (16.70 ± 7.90 µm) (Figure S1B). Interestingly, colistin crystals can be observed on the ALG@CLS MPs surface with a particle size distribution of 0.785 ± 0.35 µm and 0.430 ± 0.14 µm size (major and minor axis, respectively), i.e., recrystallized CLS is some 175 times smaller compared to raw CLS particles of Figure 2A.

**Figure 3:**
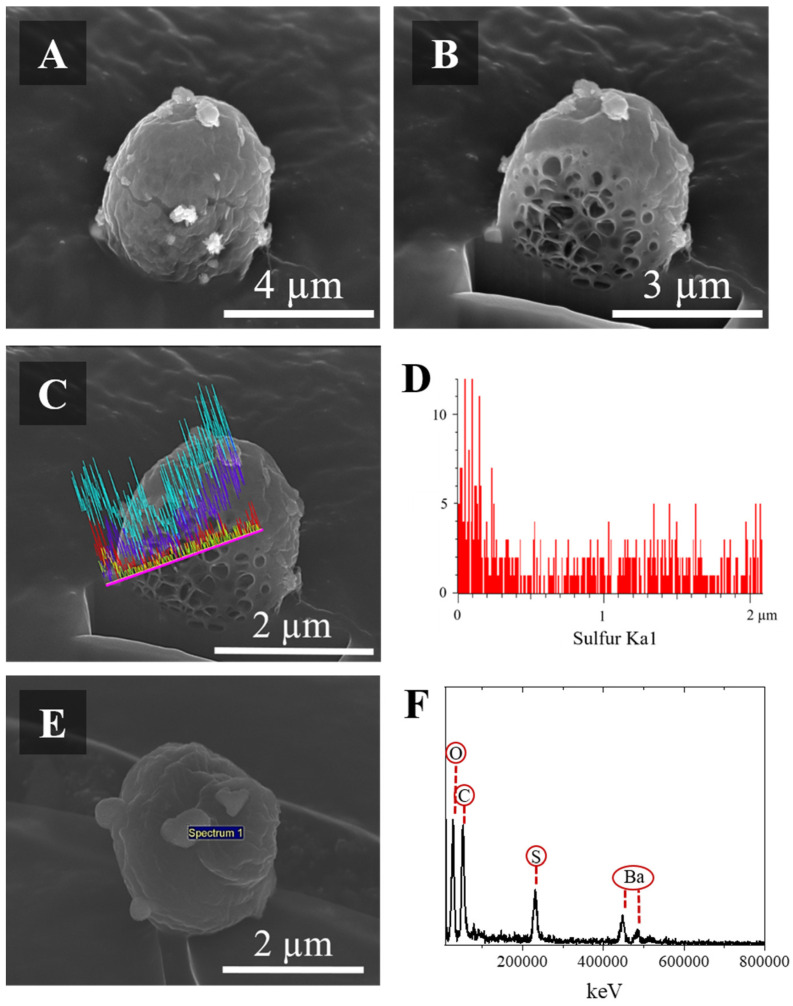
Alginate microparticles with colistin sulfate images from Dual-Beam microscope: (**A**,**E**) Intact ALG@CLS microparticles; (**B**) View of internal structure of alginate microparticles after cross-sectioning with Dual-Beam; (**C**) Internal analysis of composition along the trajectory marked with a pink line in the figure (colour codes are as follows: Blue = Oxygen, Yellow = Barium, Purple = Nitrogen, Red = Sulfur); (**D**) Detail of Sulfur profile, with a high concentration at the surface of the particle; (**F**) EDX spectrum of a CLS crystal on the surface.

To confirm the preferential deposition of CLS crystals at or near the surface of the alginate microparticles, the Dual-Beam microscope was used to observe the internal structure of alginate-based microparticles and analyze the variation of chemical composition along the radius using EDX analysis. Sulfur (S) was present in raw colistin sulfate and absent in alginate, so it could be used to monitor the concentration of the drug throughout the alginate-based microparticles. Our results show that S in the crystals is found at the surface of the ALG@CLS MPs (Table S2 and Figure S2). By contrast, in the case of ALG MPs without CLS crystals on their surface, S was not detected (Figure S2). Furthermore, microparticles were cut with a focused electron and ion beam and these internal cross-sections showed a porous internal structure without drugs inside (Figure 3). The porous internal structure was present in both types of microparticles, with and without the drug (Figures S3 and S4). The EDX analysis of the composition of the cross-section of ALG@CLS MPs revealed that the S peak only increases at the MP’s surface, coinciding with the presence of CLS crystals on the surface that was positively identified by their EDX spectrum (Figure 3F).

Both alginate-based microparticles (with and without CLS) displayed negative Z potential unlike the raw drug, whose value was positive (Table 1). Alginate microparticles had more negative potential than ALG@CLS MPs, since the –COO^−^ groups present on the alginate surface bind to the NH_3_^+^ groups from colistin sulfate. The drug loading (% DL) of the CLS in the alginate microparticles was 8.5 ± 1.5% (Table 1).

**Figure 4:**
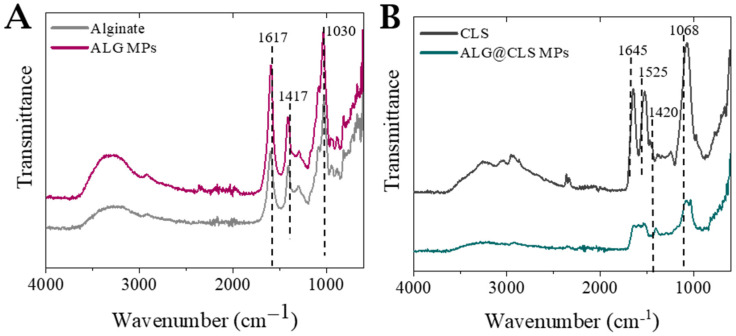
FTIR spectra of: (**A**) Raw alginate and Alginate MPs; (**B**) Raw Colistin Sulfate and ALG@CLS MPs.

Potential molecular interactions between colistin sulfate and alginate in microparticles were analyzed by FTIR (Figure 4). In the spectra of alginate, the peaks around 1617 cm^−1^ and 1417 cm^−1^ correspond to asymmetric and symmetric stretching peaks of carboxylate salt groups (-COO^−^ groups). The peak around 1030 cm^−1^ is attributed to its saccharide structure (C-O-C stretching) [33]. The same peaks are observed for alginate microparticles. For raw colistin sulfate, three main bands were identified at 1645 cm^−1^ and 1525 cm^−1^, characteristic of the amine I (C=O stretching) and amine II (N-H bending), respectively. Related to the C-N stretching, a peak at 1068 cm^−1^ was detected [4]. In the case of Alg@CLS MPs, the spectrum changed significantly compared to raw alginate and colistin sulfate. The peaks that correspond to carboxylate salt groups from alginate and amine I and II from colistin sulfate were not observed, which could be explained due to an interaction between both groups. Previous studies have shown the creation of a new peak at 1420 cm^−1^ due to the interaction between –COO^−^ of the alginate and the NH_3_^+^ groups of chitosan [34], this peak was observed in Alg@CLS MPs (Figure 4B). Therefore, the interaction between alginate and colistin sulfate could be confirmed by FTIR.

**Figure 5:**
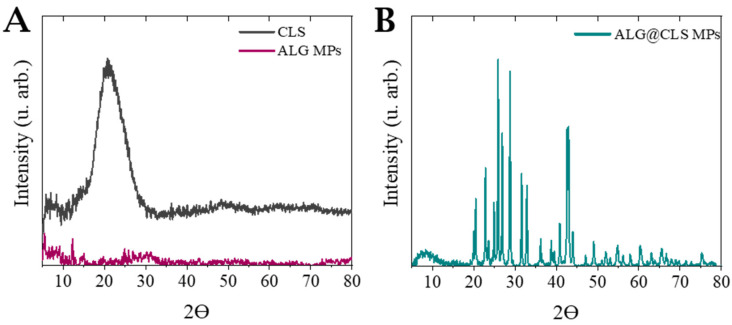
XRD patterns for: (**A**) Alginate microparticles and raw colistin sulfate; (**B**) ALG@CLS MPs.

Finally, powder XRD patterns indicated that raw colistin sulfate and alginate microparticles were amorphous according to the absence of sharp peaks. However, the process of CLS loading in alginate microparticles produced their dissolution and recrystallization, transforming them from an amorphous form to crystalline as evinced by sharp diffraction peaks (Figure 5B) [35]. The crystallization process has been studied in other drugs such as carbamazepine [36], ibuprofen [37] or hydrocortisone acetate [38]. In this case, the evidence from microscopy analysis shows that the crystals are present at the external surface of the alginate microparticles, a fact that suggests that heterogeneous nucleation at the surface is likely the initiating step for this process.

The loading of CLS as a crystalline material offers the advantages of higher physical and chemical stability in contrast with the pure amorphous form [39,40]. As already mentioned, the amorphous form of this drug is able to absorb an important amount of water when it was exposed to high relative humidity, leading to a decrease in the amount of breathable drug and increasing moisture-induced deterioration [12]. In general, crystalline properties are preferred, because of their higher levels of purity and stability [41], although amorphous forms have the advantage of faster solubility. The structure synthesized, in this case, presents several important advantages: (i) a support structure of a size suitable for inhalation, especially suited to pulmonary delivery thanks to the mucoadhesive properties of alginate; (ii) a high load (>8.5%) of the CLS drug; (iii) the drug is distributed in small, micron-sized crystalline particles on the surface of the alginate microparticles. This small size and periphery distribution allow a rapid dissolution of the drug, as shown next.

### 3.3. In Vitro Release

Drug-release experiments were done in PBS and showed that 100% of the drug was released in 4 h, with ca. 70% released after one hour. Parameter estimation was performed according to the description given in Materials and Methods (Section 2.8). The main results are summarized in Table 2.

Table 2 shows the results for the three parameters estimated (D, k and m) and the statistical analysis of the fitting. The value estimated for D is in accordance with previous results published for other antibiotics such as Amtyl, Ciprotyl or Trisullak, that have diffusion coefficients around 8 × 10^−9^ m^2^/s [12]. Additionally, the value calculated for the Intrinsic dissolution rate constant (k) is also in the range of previous results published for other drugs. An example was described by Skinner et al. [41] whose crystalline drug, josamycin, has an intrinsic dissolution constant (5.1 × 10^8^ 1/m^2^s) very similar to crystalline colistin sulfate.

**Figure 6:**
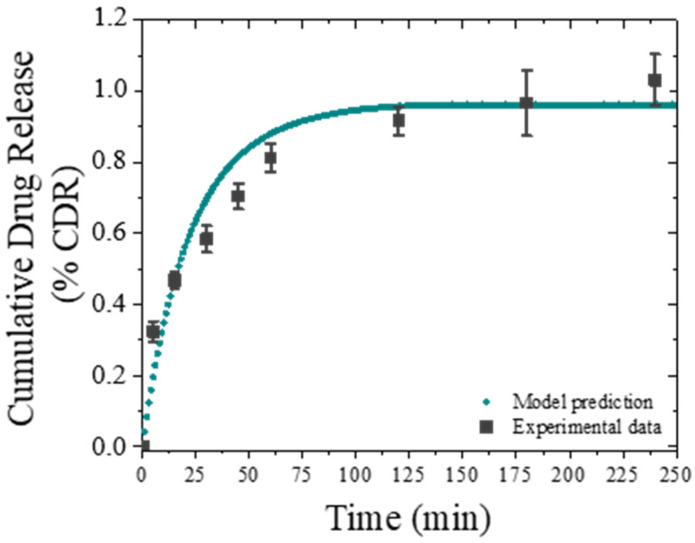
Cumulative Drug Release: Drug release versus time in PBS and the model prediction after parameter estimation (blue line); (mean ± SD, *n* = 3).

Figure 6 exhibits the cumulative drug release (% CDR) of alginate microparticles at 37 °C versus time, and the model prediction with the parameters reported in Table 2. The value of the weighted residuals is less than the chi-square value (X^2^), and therefore, it passes this test. The F-value is also less than F-critical and therefore, the lack of fit test results are positive as well. Therefore, it can be concluded that the model accurately approximates the experimental data obtained for drug release.

### 3.4. Antimicrobial Activity against Pseudomonas aeruginosa

The study of antimicrobial activity was performed by measuring the optical density (OD600) of bacteria treated with raw colistin sulfate and alginate microparticles with CLS. The results of the antimicrobial activity were confirmed with the agar dilution method (Figure S5). The MIC of raw colistin sulfate against planktonic *P*. *aeruginosa* was 0.5–1 µg/mL and the MBC was >4 µg/mL. The formulation of CLS with alginate microparticles exhibited the same MIC and MBC that colistin sulfate alone [9]. These results show that the encapsulation process does not affect the antibacterial effects of CLS against this bacteria strain.

**Figure 7:**
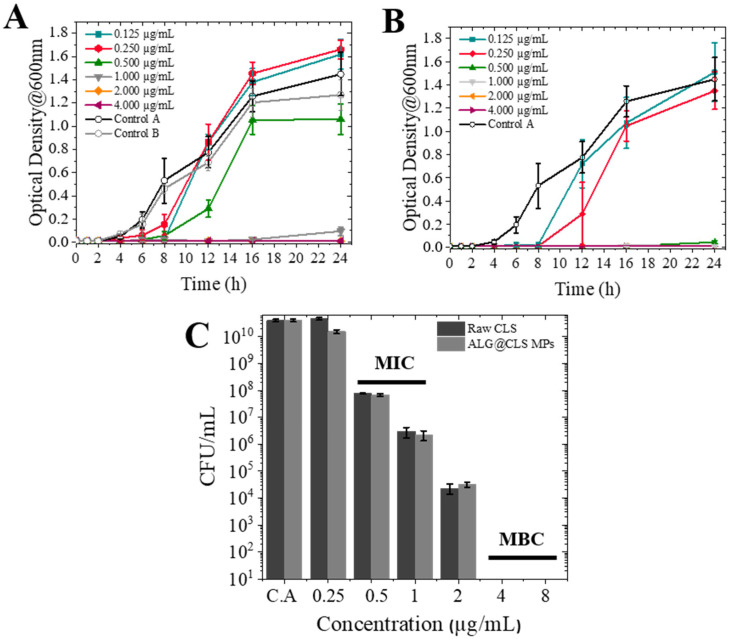
Antibacterial activity of colistin sulfate against *P. aeruginosa*. OD600 versus total concentration of (**A**) Raw colistin sulfate; (**B**) Colistin sulfate encapsulated in ALG@CLS MPs (control A was bacteria untreated and control B was bacteria in contact with water); (**C**) CFU/mL of *P. aeruginosa* (mean ± SD, *n* = 3; *p* < 0.05). The concentrations (µg/mL) refer to the amount of CLS, either free or encapsulated in the ALG MPs.

Figure 7A,B presents the growth curves of *P*. *aeruginosa* once in contact with the treatment of colistin sulfate and ALG@CLS MPs. The curves showed a reduction in bacterial growth when colistin sulfate concentration was increased. When *P*. *aeruginosa* was treated with ALG@CLS MPs, a delay is observed in the appearance of the log phase at 0.250 µg/mL concentration. In contrast, this delay was observed with a somewhat higher concentration (0.500 µg/mL) with free CLS. These results indicate that a lag phase up to 8 h was present, showing that the growth was repressed and the log phase did not start until 12 h from the beginning of the culture and that *P*. *aeruginosa* was slightly more susceptible to the microparticles than raw colistin sulfate. In agreement with these results, the MIC concentration was set at 0.500–1 µg/mL because at this concentration bacterial growth was inhibited. MBC value was fixed as 4–8 µg/mL, which were the concentrations found to inhibit the growth bacteria. These concentrations were confirmed with the agar dilution method (Figure 7C).

**Figure 8:**
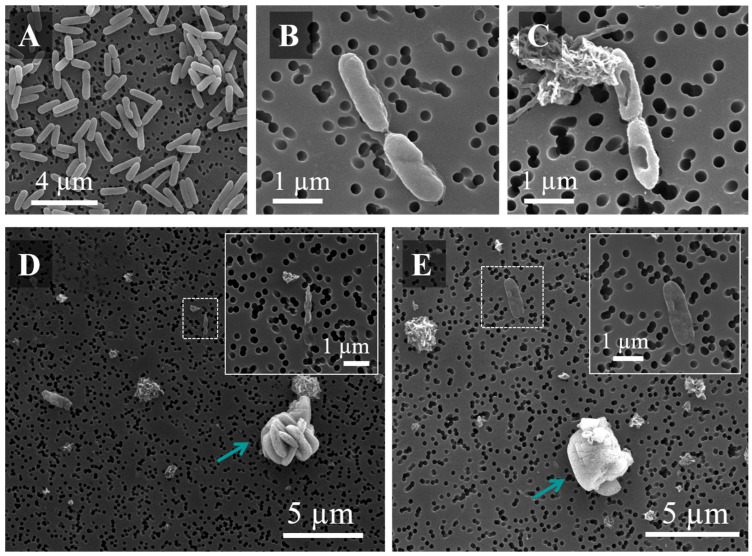
Images obtained by SEM of *P. aeruginosa*: (**A**) Control; (**B**) After treatment with raw colistin sulfate; (**C**) After treatment with ALG@CLS MPs; (**D**,**E**) In contact with ALG@CLS MPs (blue arrow) with dead bacteria in contact with CLS-loaded microparticles.

The morphological changes of *P*. *aeruginosa* following treatment were also documented in depth by SEM observations. Bacteria control without treatment by either raw colistin sulfate or CLS-loaded microparticles revealed a smooth and non-damage cell morphology (Figure 8A). In contrast, when bacteria were treated with the drug or with ALG@CLS MPs, after 24 h incubation, strong changes were observed in the bacteria morphology (Figure 8B,C). The membrane integrity of *P*. *aeruginosa* was affected by colistin sulfate (supplied either as free CLS or loaded in microparticles) with holes and deformations clearly shown on the membrane. In Figure 8C,D, it could be observed that when bacteria are in the same growth culture medium as CLS-loaded alginate microparticles at a concentration over MIC, the bacteria died. CLS was released from ALG MPs, displaying its antimicrobial activity. Finally, the SEM images suggested that colistin sulfate treatment may inhibit cell wall formation. There are two possible targets, the cytoskeletal system or the enzyme (PBP2), both of which are necessary for peptidoglycan synthesis and bacteria cell elongation [9].

### 3.5. Cytotoxicity Assay

**Figure 9:**
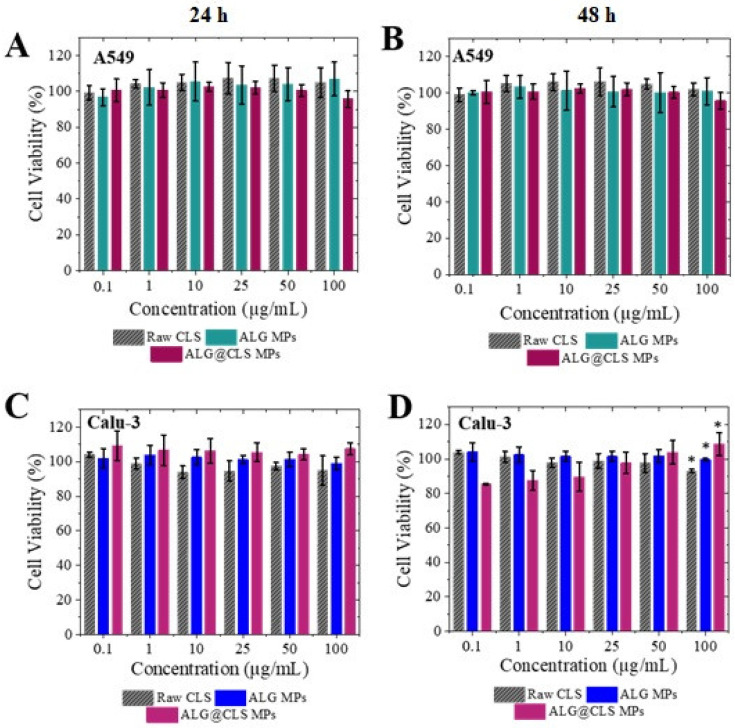
Cytotoxicity of free drug and alginate microparticles. Graphs indicate cell viability at two different times. A549 (**A**) 24 h and (**B**) 48 h. Calu-3 (**C**) 24 h and (**D**) 48 h. Notes: * Significant differences (*p* < 0.05) between samples studied compare to raw colistin sulfate (mean ± SD, *n* = 3).

The effect of alginate microparticles (with and without CLS) and raw colistin sulfate on the relative cell viability of A549 and Calu-3 cell lines are shown in Figure 9. Both cell lines were placed in contact for 24 h and 48 h at a concentration ranging from 0.1–100 µg/mL.

Alginate microparticles were considered biocompatible in both cell lines, showing their safety for their administration until 100 µg/mL in relation to the international standard ISO 10993-5. The samples treated with alginate microparticles loaded with colistin sulfate have similar cell viability to those treated with microparticles free of drug and blank microparticles. No sample tested showed a significant suppressive effect on A549 and Calu-3 viability, the viability values obtained were higher than 70% in all cases, even at concentrations of 100 µg/mL, indicating that there is no apparent cytotoxicity in epithelial cells used. It must stress that the maximum concentration analyzed was 100 µg/mL, ca. 25 times the MBC (4 µg/mL) for *P*. *aeruginosa*. In this way, the administration of these microparticles will make it possible to eliminate the infection with this bacteria strain by using a concentration clearly above the MBC, with no seeming cytotoxicity to human airway cells.

## 4. Conclusions

A process combining atomization, sieving and drying stages can be used to generate CLS-loaded alginate microparticles with a size suitable for direct administration by inhalation and high drug loading (>8%). The delivery profile from these microparticles showed a fast release over 4 h in PBS, suggesting that it may be useful in the treatment of bacterial lung infections. SEM, FTIR, EDX and PXRD characterization revealed that microparticle formation was successful with colistin sulfate deposits present as approximately 1 micron crystals on the surface of alginate microparticles promoting rapid drug dissolution. The internal structure of the microparticles showed porosity and the absence of drugs inside the particles, CLS being present only on the surface. These microcrystal-loaded particles displayed a strong antibacterial activity against *Pseudomonas aeruginosa* inhibiting growth at concentrations comparable to or even lower than the raw drug. Finally, cytotoxicity assays in alveolar (A549) and bronchial (Calu-3) cell lines of colistin sulfate loaded in alginate microparticles revealed a high biocompatibility, comparable to that of free colistin sulfate. In summary, this formulation offers high potential treatment for direct delivery by inhalation in respiratory infections.

## Figures and Tables

**Table 1:** Characterization of drug- and alginate-based microparticles (mean ± SD, *n* = 150).

	SEM					Zeta Potential (mV)	% DL	% EE
Samples	Mean ± SD (µm)	D 0.1 (µm)	D 0.1 (µm)	D 0.1 (µm)	Span			
Colistin sulfate	173.30 ± 47.5	108.97	177.05	229.97	0.68	7.37 ± 0.9	-	-
Alginate MPs	4.80 ± 1.6	2.87	4.62	6.76	0.84	−35.14 ± 0.4	-	-
Alg@CLS MPs	4.45 ± 1.4	2.80	4.35	6.48	0.85	−14.14 ± 0.1	8.50 ± 1.5	28.80 ± 1.1

**Table 2:** Parameters estimated from drug-release modelling.

Parameters Estimated			Goodness of Fit Test		Lack of Fit Test	
D (m^2^/s)	K (1/m^2^s)	m	Weighted Residuals	X^2^	F-Value	F-Critical
7.84 × 10^−9^	2.35 × 10^8^	1.81 × 10^−9^	10.98	11.07	0.069	3.326
Confidence interval 95%						
D (7.73 × 10^−9^–7.89 × 10^−9^)			k (2.30 × 10^8^–2.40 × 10^8^)		m (1.77 × 10^−9^–1.85 × 10^−9^)	

## Data Availability

Not applicable.

## Supplementary Materials

The following supporting information can be downloaded at: https://www.mdpi.com/article/10.3390/pharmaceutics14122763/s1, Figure S1: Optical and SEM microscopes images of alginate microparticles; (A,B) Alginate 2% *w*/*v*; (C,D) Alginate 2.5% *w*/*v*; Table S1: Size of alginate microparticles; Table S2: EDX analysis; Figure S2: EDX analysis. SEM and Dual-Beam images and spectrums: (A) Raw colistin sulfate; (B) EDX of raw colistin sulfate; (C) Alginate microparticles; (D) EDX of alginate microparticles; Figure S3: Alginate microparticles with colistin sulfate images from Dual-Beam microscope: (A) Initial ALG@CLS MPs; (B,C) Internal structure of microparticles after two cuts; Figure S4: Alginate microparticles without drug, images from Dual-Beam microscope: (A) Initial alginate microparticle; (B–D) Internal structure of microparticles after three cuts; (E,F) Internal analysis composition of microparticle (oxygen, carbon, barium and sulfur); Figure S5: Protocol to determinate MIC and MBC concentrations of colistin sulfate against *P. aeruginosa.*
