# Supplementary material for: Engineering Alginate-Based Dry Powder Microparticles to a Size Suitable for the Direct Pulmonary Delivery of Antibiotics
Source: Pharmaceutics. 2022 Dec 9;14(12):2763. doi: 10.3390/pharmaceutics14122763 (PMC9781482; doi:10.3390/pharmaceutics14122763)
Supplement: Supplementary file 1 [file pharmaceutics-14-02763-s001.zip › pharmaceutics-2019190-supplementary.pdf]

pharmaceutics

Supplementary Materials: Engineering alginate-based dry powder microparticles to a size suitable for direct pulmonary delivery of antibiotics

Beatriz Arauzo, Álvaro González-Garcinuño, Antonio Tabernero, Javier Calzada-Funes, María Pilar Lobera, Eva M. Martín del Valle, Jesús Santamaria

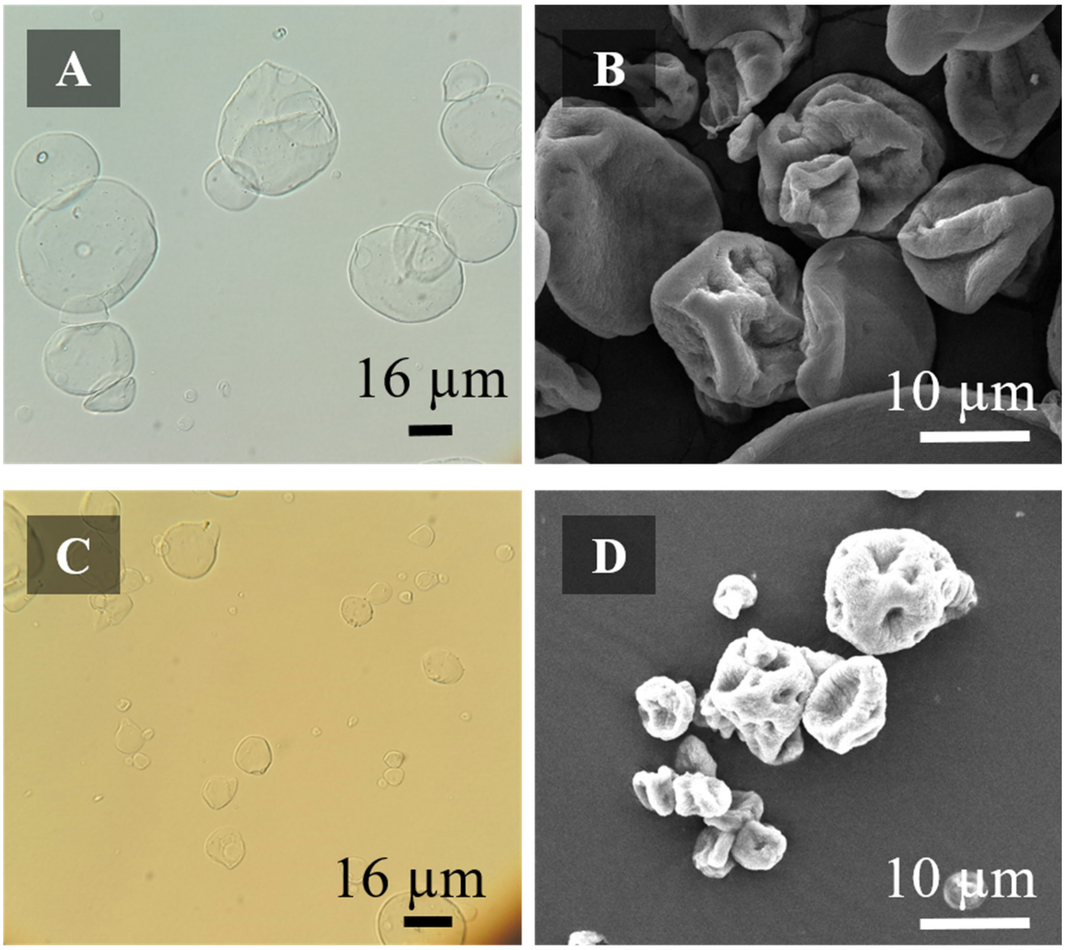

Figure S1. Optical and SEM microscopes images of alginate microparticles; (**A** - **B**) Alginate 2% w/v; (**C** - **D**) Alginate 2.5% w/v.

Table S1. Size of alginate microparticles.

Samples	Optical microscope (μm) (in water)	SEM (μm) (as powder)
**Alginate 2% w/v MPs**	36.10 ± 14.50	11.53 ± 5.02
**Alginate 2.5% w/v MPs**	16.70 ± 7.90	7.57 ± 4.18

Table S2. EDX analysis.

	Raw Colistin		Alginate Microparticles	
**Element**	**Weight%**	**Atomic%**	**Weight%**	**Atomic%**
**C K**	44.03	54.18	78.92	82.58
**N K**	18.32	19.33	7.76	6.96
**O K**	19.74	18.24	13.32	10.47
**S K**	17.90	8.25	-	-
**Ba L**	-	-	-	-
**Total**	100.00		100.00	

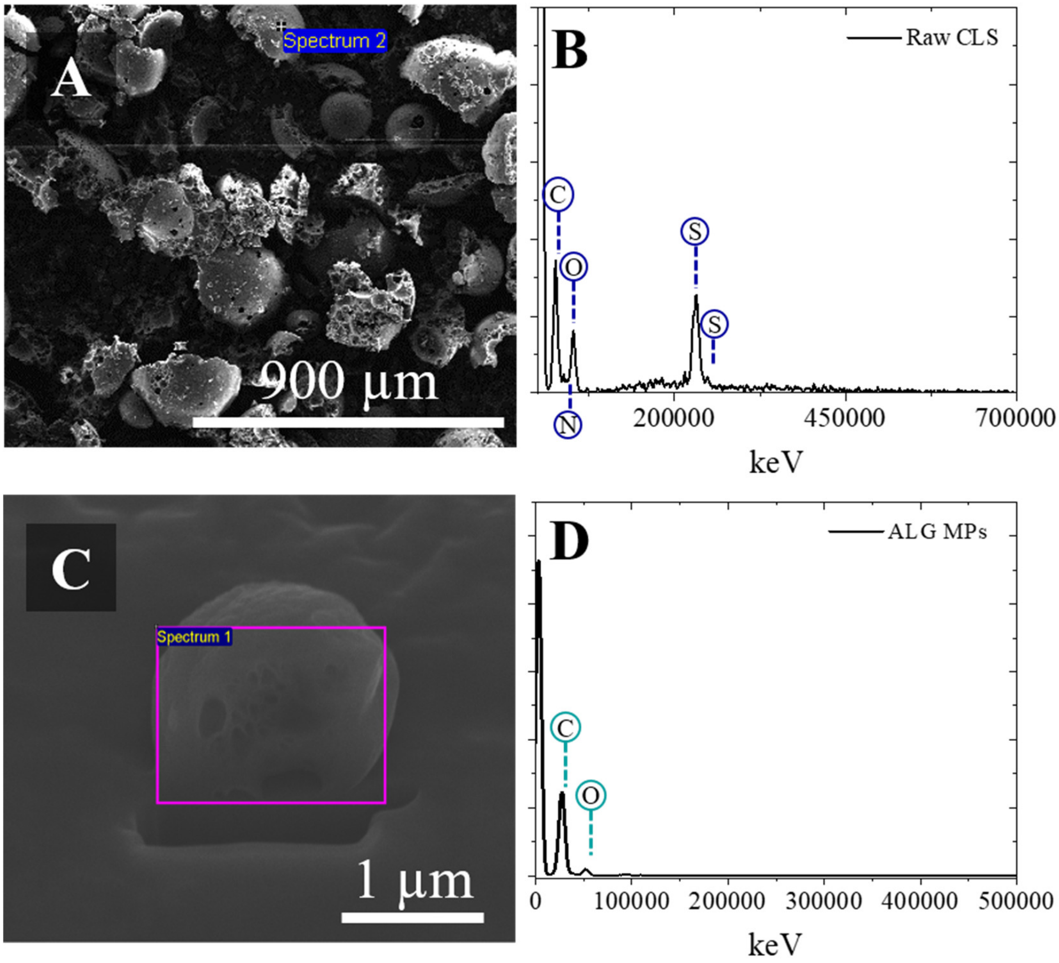

Figure S2. EDX analysis. SEM and Dual-Beam images and spectrums: (**A**) Raw colistin sulfate; (**B**) EDX of raw colistin sulfate; (**C**) Alginate microparticles; (**D**) EDX of alginate microparticles.

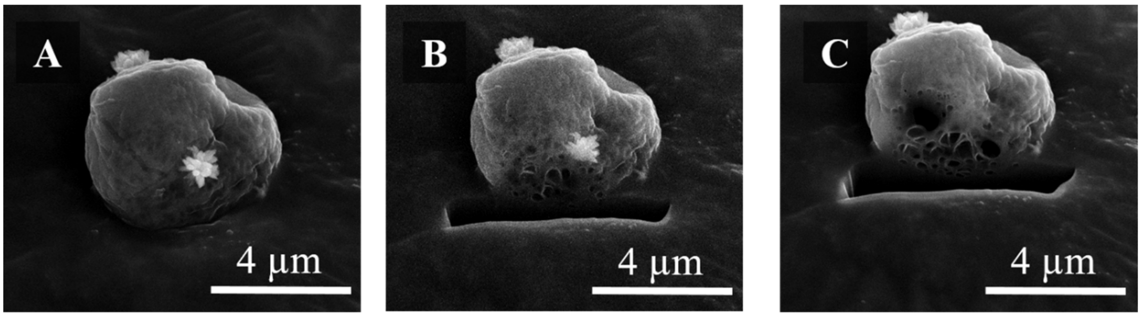

Figure S3. Alginate microparticles with colistin sulfate images from Dual-Beam microscope: **(A)** Initial ALG@CLS MPs; **(B - C)** Internal structure of microparticles after two cuts.

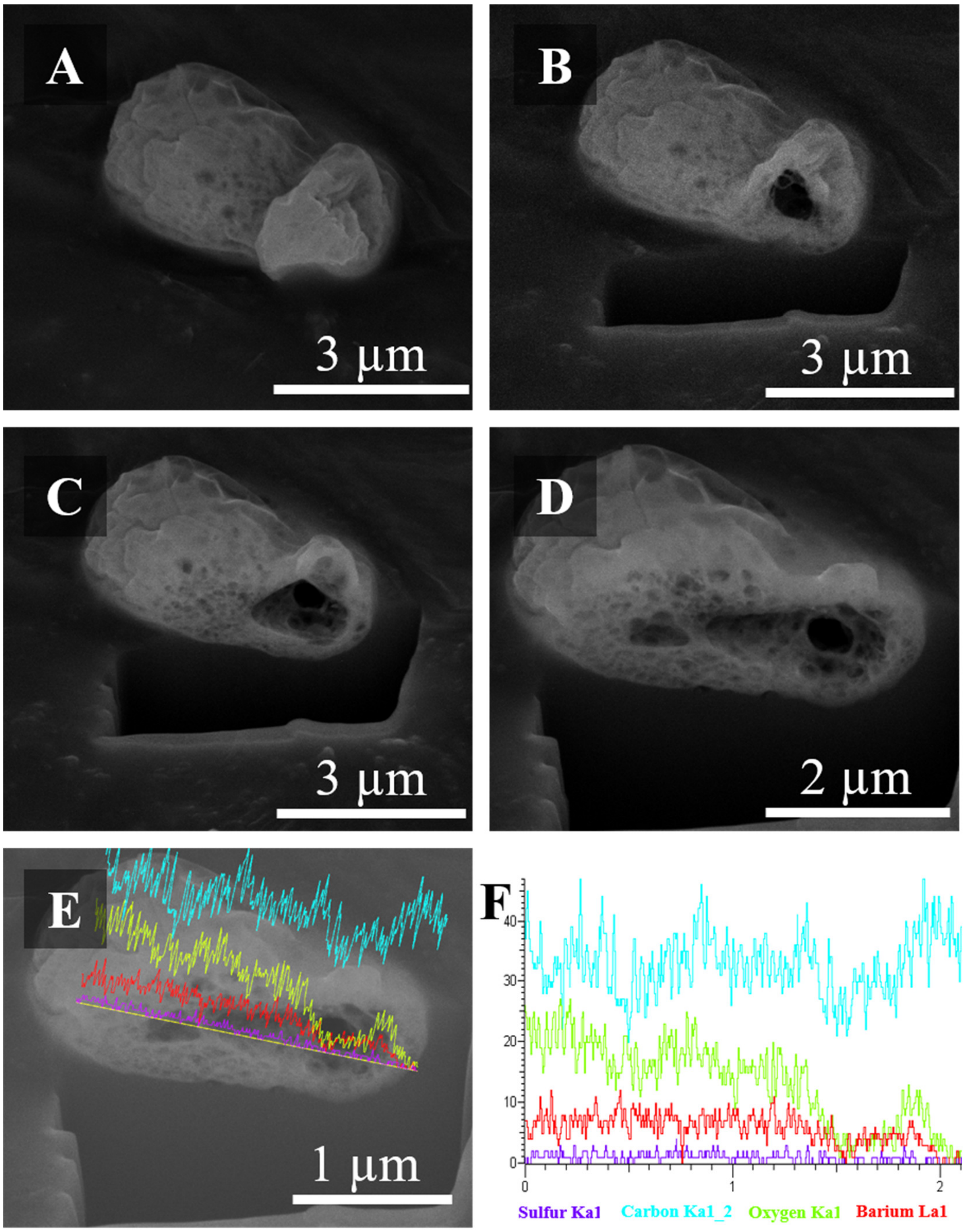

Figure S4. Alginate microparticles without drug, images from Dual-Beam microscope: (**A**) Initial alginate microparticle; (**B** - **D**) Internal structure of microparticles after three cuts; (**E** - **F**) Internal analysis composition of microparticle (oxygen, carbon, barium and sulfur).

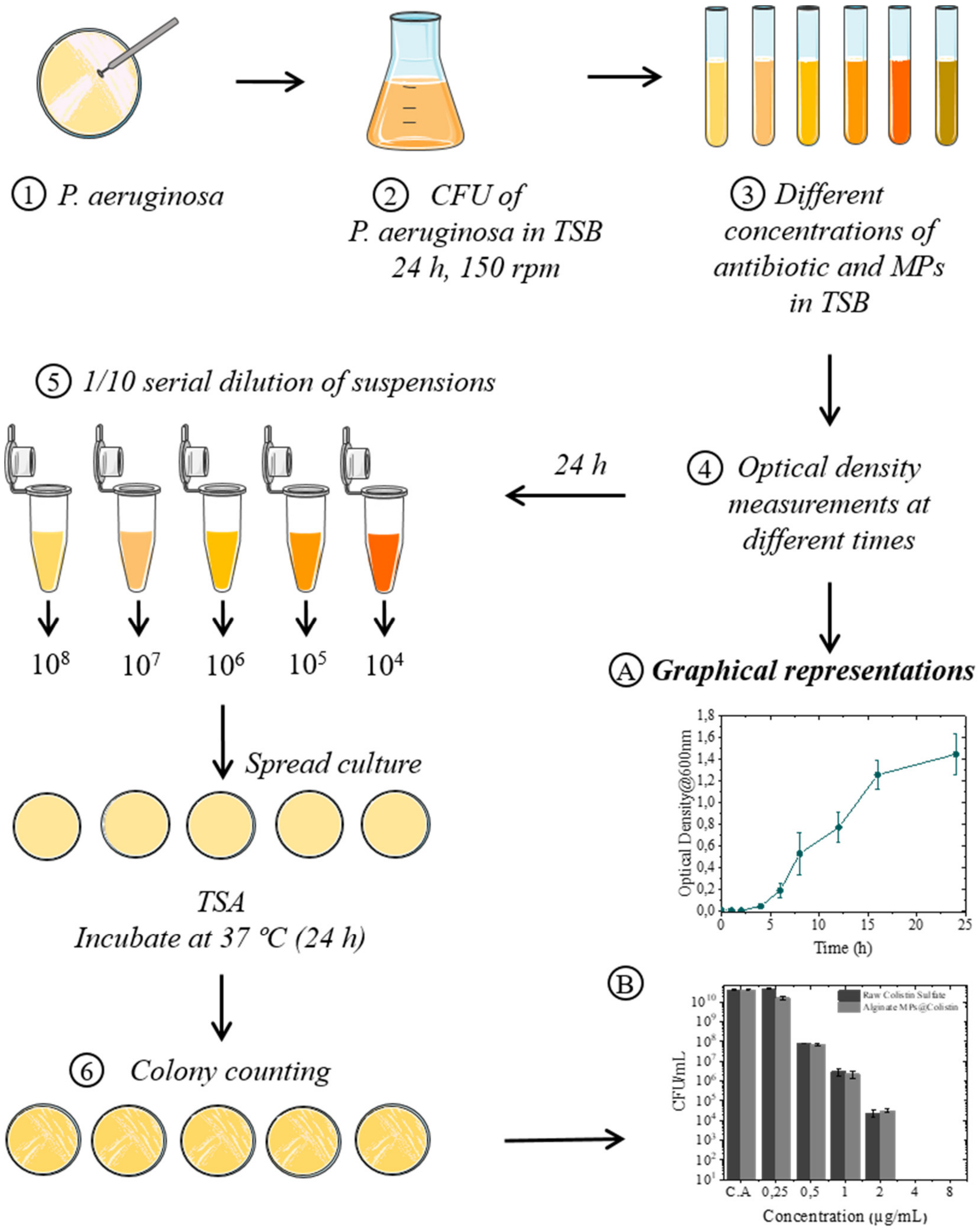

Figure S5. Protocol to determinate MIC and MBC concentrations of colistin sulfate against *P. aeruginosa*.
